# A new perspective on the therapeutic potential of tumor metastasis: targeting the metabolic interactions between TAMs and tumor cells

**DOI:** 10.7150/ijbs.99680

**Published:** 2024-09-23

**Authors:** Xuan Zhao, Tong Ren, Sijin Li, Xu Wang, Rui Hou, Zhangchun Guan, Dan Liu, Junnian Zheng, Ming Shi

**Affiliations:** 1 Cancer Institute, Xuzhou Medical University, China.; 2 Center of Clinical Oncology, The Affiliated Hospital of Xuzhou Medical University, China.; 3 Jiangsu Center for the Collaboration and Innovation of Cancer Biotherapy, Xuzhou Medical University, China.; 4 College of Pharmacy, Xuzhou Medical University, Xuzhou, Jiangsu, China.

**Keywords:** Tumor-associated macrophages, Tumor cells, Epithelial-mesenchymal transition, Metabolism, Tumor microenvironment

## Abstract

Tumor-associated macrophages (TAMs) undergo metabolic reprogramming, encompassing glucose, amino acid, fatty acid metabolism, tricarboxylic acid (TCA) cycle, purine metabolism, and autophagy, within the tumor microenvironment (TME). The metabolic interdependencies between TAMs and tumor cells critically influence macrophage recruitment, differentiation, M2 polarization, and secretion of epithelial-mesenchymal transition (EMT)-related factors, thereby activating intratumoral EMT pathways and enhancing tumor cell invasion and metastasis. Tumor cell metabolic alterations, including hypoxia, metabolite secretion, aerobic metabolism, and autophagy, affect the TME's metabolic landscape, driving macrophage recruitment, differentiation, M2 polarization, and metabolic reprogramming, ultimately facilitating EMT, invasion, and metastasis. Additionally, macrophages can induce tumor cell EMT by reprogramming their aerobic glycolysis. Recent experimental and clinical studies have focused on the metabolic interactions between macrophages and tumor cells to control metastasis and inhibit tumor progression. This review highlights the regulatory role of TAM-tumor cell metabolic codependencies in EMT, offering valuable insights for TAM-targeted therapies in highly metastatic tumors. Modulating the metabolic interplay between tumors and TAMs represents a promising therapeutic strategy for treating patients with metastatic cancers.

## Introduction

The tumor microenvironment (TME) hosts a variety of immune cells, such as macrophages, lymphocytes, neutrophils, and myeloid suppressor cells. Macrophages, originating from bone marrow monocytes, dominate, constituting 30-50% of these immune cells 1. These macrophages are classified into two main subtypes: M1 macrophages, which effectively eradicate infectious microorganisms and exhibit strong tumoricidal properties, and M2 macrophages, which facilitate tumor epithelial-mesenchymal transition (EMT) and progression 2. The metabolic reprogramming of tumor-associated macrophages (TAMs) results in the secretion of various cytokines that advance cancer progression by inducing EMT, angiogenesis, metabolic reprogramming, multidrug resistance (MDR), and cancer stem cell (CSC) traits 3.

EMT is a complex, dynamic process where epithelial cells transform into a mesenchymal phenotype, significantly driving tumor metastasis. Several extracellular signaling factors are essential in inducing EMT in tumor cells. Metabolically reprogrammed M2-type TAMs secrete EMT-promoting factors, engage in various signaling pathways, and modulate marker expression—down-regulating epithelial markers and up-regulating mesenchymal markers—thus transforming tumor cells from an epithelial to a mesenchymal phenotype 4, 5.

The interaction of numerous metabolic pathways between TAMs and tumor cells underscores the pivotal role of EMT in cancer progression. The metabolic interaction between tumor cells and TAMs in the TME, along with their intrinsic changes, are critical drivers of tumor progression. The metabolic reprogramming of TAMs and tumor cells is a dynamic, reversible process in cancer progression, involving elements such as metabolic pathways and metabolites. Some of these elements have been utilized in the design and clinical application of targeted drugs.

This review summarizes the regulation of EMT through metabolic codependencies between TAMs and tumor cells, including: (1) the metabolic reprogramming of macrophages in the TME-promoting tumor EMT, (2) the metabolic microenvironment shaped by tumor cells regulating macrophage EMT-promoting activity, (3) macrophages inducing EMT in tumor cells *via* metabolic reprogramming, and (4) targeting metabolic interactions between TAMs and tumor cells to treat metastatic tumors. To date, various potential therapeutic strategies targeting the metabolic codependencies between TAMs and tumor cells have been confirmed, providing a basis for developing novel clinical anti-tumor combination therapies targeting TAMs and new anti-tumor drugs.

## Metabolic reprogramming of TAMs promotes EMT in cancer cells

**Figure 1:**
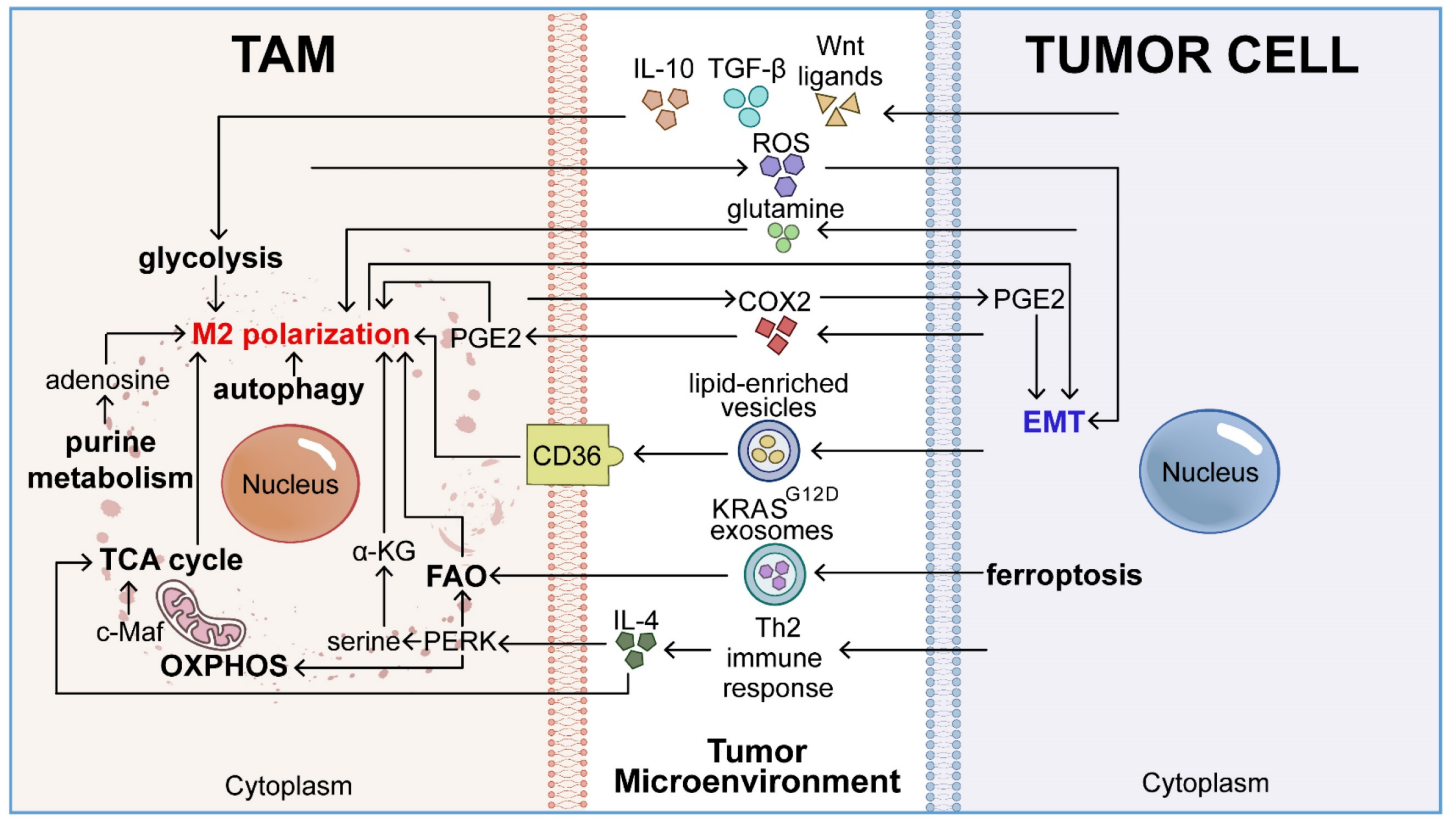
Metabolic reprogramming of TAMs within the TME enhances their pro-EMT potential. TAMs in the TME undergo diverse metabolic transitions, including glucose utilization, fatty acid catabolism, amino acid metabolism, TCA cycle activities, purine metabolism, aerobic respiration, and autophagy. Such metabolic reconfiguration enables TAMs to shift towards a tumor-promoting M2 phenotype, thereby amplifying their capacity to facilitate EMT.

Metabolic reprogramming, a hallmark of cancer, endows macrophages with tumor-promoting properties. Tumors actively reprogram macrophage metabolism—including glucose, fatty acid, and amino acid metabolism, the TCA cycle, purine metabolism, aerobic metabolism, and autophagy—through metabolites, cytokines, and other signaling mediators. This metabolic reprogramming enhances the recruitment, M2 polarization, and secretion of EMT-related factors by macrophages, thereby promoting tumor EMT and progression (**Figure 1**).

### Glucose metabolism

Macrophages are the primary cell population responsible for glucose uptake and metabolism within the TME. Enhanced glucose metabolism in TAMs significantly contributes to the accumulation of tumor metabolites. For instance, lactate and succinate exhibit potent tumor-promoting effects and promote O-GlcNAcylation of lysosomal cathepsin B *via* O-GlcNAcylation transferase (OGT), thereby facilitating tumor metastasis 6, 7. In the early stages of pancreatic tumor development, the inflammatory hyperglycemic microenvironment profoundly influences macrophage-mediated EMT in malignant tumors 8. Under hyperglycemic conditions, benign and premalignant pancreatic ductal epithelial cells (PDEC) exhibit glucose- and macrophage-dependent EMT triggers in a coculture system. This induction is associated with the upregulation of EMT inducers such as interleukin (IL)-6 and tumor necrosis factor-alpha (TNF-α), along with EMT transcription factors, promoting the downregulation of the epithelial marker E-cadherin 8. Pancreatic ductal adenocarcinoma (PDAC) cells specifically induce DNA methylation and downregulation of glucose metabolism and oxidative phosphorylation (OXPHOS) genes in M1 macrophages, reprogramming them into M2 macrophages to support tumor growth, EMT, and metastasis 9.

Glycolysis is a key pathway of glucose metabolism, and the glycolytic metabolism of macrophages has bidirectional effects on tumorigenesis, exhibiting both tumor-promoting and tumor-inhibiting properties. The aerobic glycolysis in M1 macrophages promotes phagocytosis through reactive oxygen species (ROS) production. Conversely, the glycolytic metabolism of M2 macrophages proceeds at a slower rate under aerobic conditions. M2 macrophages suppress ROS and nitric oxide (NO) production by blocking the glycolysis/pentose phosphate pathway (PPP)/ nicotinamide adenine dinucleotide phosphate (NADPH)/ROS and TCA cycle/L-arginine/ inducible nitric oxide synthase (iNOS)/NO pathways, thereby attenuating the tumoricidal functions of macrophages 10. Tumor cells can trigger M2 polarization and pro-EMT activities in macrophages by secreting various factors that induce macrophage glycolysis. Tumor-derived soluble factors, such as hyaluronan fragments, promote glycolysis in TAMs by upregulating the glycolytic enzyme 6-phosphofructo-2-kinase/fructose-2,6-biphosphatase 3 (PFKFB3) 11. IL-10, transforming growth factor beta (TGF-β), and Wnt ligands from hepatocellular carcinoma (HCC) cells upregulate Wnt2b expression and promote M2 polarization in TAMs by activating the Wnt2b/β-catenin/c-Myc signaling axis and glycolysis 12. Furthermore, the aerobic glycolysis exhibited by tumor-conditioned macrophages promotes angiogenesis, tumor cell extravasation, EMT, and metastasis in PDAC 13.

### Fatty acid metabolism

Fatty acid metabolism is essential for macrophage M2 polarization, with tumor cells manipulating this process to induce a pro-EMT phenotype in macrophages.

#### Fatty acid intake and oxidation

The scavenger receptor CD36, upregulated in metastasis-associated macrophages (MAMs), plays a significant role in promoting liver metastasis. Deleting CD36 in MAMs markedly reduces liver metastasis in mice. In patients with liver metastases, high CD36 expression correlates with increased M2 MAM infiltration. These MAMs accumulate lipid droplets and uniquely phagocytose long-chain fatty acids from tumor cells, which are transported *via* extracellular vesicles. These lipid-enriched vesicles are selectively internalized by macrophages through the CD36 receptor, providing the energy necessary for macrophage tumor-promoting activities 14. IL-4 in the TME induces both macrophage M2 polarization and fatty acid uptake and oxidation 10. Oxidative stress prompts PDAC cells to secrete the mutant KRAS (KRAS^G12D^) protein *via* autophagy-dependent ferroptosis. KRAS^G12D^ is then transferred to macrophages through exosomes, leading to M2 polarization *via* a STAT3-dependent FAO mechanism 15. Recent research also highlights the role of fatty acid binding protein 4 in transferring saturated fatty acids to induce macrophage pyroptosis, mediating the NOD-like receptor thermal protein domain-associated protein 3 (NLRP3)/IL-1β axis, further regulating EMT, and enhancing pancreatic cancer cell metastasis in patients with obesity 16.

#### Arachidonic acid metabolism

Arachidonic acid, a polyunsaturated fatty acid, plays a pivotal role in the TME. Exosomes released from PDAC cells, such as AsPC-1, increase their fusion rate with THP-1-derived macrophages through the mediation of arachidonic acid, switching these macrophages to the M2 phenotype and triggering the secretion of pro-EMT factors, including VEGF, monocyte chemoattractant protein-1 (MCP-1), IL-6, IL-1β, matrix metalloproteinase (MMP)-9, and TNF-α 17.

Cyclooxygenase 2 (COX-2) converts arachidonic acid into the fatty acid derivative prostaglandin (PG). Primarily secreted by TAMs in the TME, elevated COX-2 expression is associated with poor prognosis in patients with breast cancer. TAM-derived COX-2 promotes its expression in breast cancer cells, thereby enhancing macrophage M2 polarization 18. PDAC cell-derived exosomes can increase Prostaglandin E2 (PGE2) production, further activating M2 polarization in macrophages 19. Macrophage-secreted TGF-β can elevate COX-2 expression by upregulating VEGF, connective tissue growth factor (CTGF), hepatocyte growth factor (HGF), fibroblast growth factor (FGF), and TNF-α. In colon cancer, COX-2 overexpression in TAMs can also upregulate TGF-β through a paracrine pathway, activating TGF-β-induced signaling pathways independent of Smads, such as the NF-kappaB (NF-κB) pathway. Furthermore, COX-2 secreted by TAMs induces PGE2 and IL-6 secretion, activating the extracellular signal-regulated kinase (ERK) 1/2, STAT3, β-catenin, and phosphatidylinositol-3-kinase (PI3K)/protein kinase B (AKT/PKB) signaling pathways. These pathways are pivotal in mediating EMT in breast, colorectal, lung, and osteosarcoma cancer cells 18, 20.

### Amino acid metabolism

Amino acid metabolism in macrophages, including the activation of glutamine catabolism and the hexosamine biosynthesis pathway, significantly influences M2 macrophage polarization.

Tumor cell-secreted glutamine induces M2 polarization in macrophages 10. Upon lipopolysaccharide (LPS) activation, macrophages exhibit increased glutamine consumption and enhanced glycine production. Glycine inhibits LPS-induced NO production and macrophage activation, while glutaminolysis is essential for inducing M2 polarization. M2 macrophages heavily depend on glutamine entry into the TCA cycle and primarily rely on fatty acid β-oxidation and TCA cycling, facilitating the conversion of L-arginine into polyamines and L-proline through arginase 1 (Arg1) to support tumor growth 21.

Helper T cell 2 cytokine IL-4 and the TME potentiate the activation of the protein kinase RNA-like ER kinase (PERK)-signaling cascade in TAMs, enhancing immunosuppressive M2 polarization and proliferation. PERK signaling promotes mitochondrial respiration and FAO to meet cellular energy demands and regulates phosphoserine aminotransferase 1 (PSAT1) activity through ATF4 to mediate the serine biosynthesis pathway. Increased serine biosynthesis enhances mitochondrial function and α-ketoglutarate (α-KG) production. Mitochondrial OXPHOS, FAO, and α-KG drive tumor EMT, metastasis, and progression by inducing M2 polarization in macrophages 22.

### TCA cycle

The TCA cycle, prevalent in TAMs, supports pro-tumorigenic bioenergetic functions and phenotypic plasticity within the macrophage population. Due to the slower rate of aerobic glycolysis, M2 macrophages sustain adenosine triphosphate (ATP) production through the TCA cycle, reducing ROS generation and compromising their tumoricidal capabilities 10. High expression of the transcription factor cellular musculoaponeurotic fibrosarcoma (c-Maf) in TAMs supports their immunosuppressive function. Additionally, c-Maf acts as a metabolic checkpoint regulating the TCA cycle and uridine diphosphate N-acetylglucosamine (UDP-GlcNAc) biosynthesis, promoting M2 macrophage polarization and activation, thereby fostering tumor cell EMT 6. IL-4-induced polarization of M2 macrophages can interchangeably utilize glucose or tumor-derived lactate as a TCA cycling carbon source. This maximizes adenosine triphosphate-citrate lyase (ACLY)-dependent histone acetylation on M2 gene-specific promoters 23.

### Purine metabolism

The lipid metabolism of TAMs can be reprogrammed to purine metabolism within the TME. Upregulation of purine metabolism characterizes TAMs with a pro-tumor and end-differentiation phenotype, correlating with poor responsiveness to immune checkpoint blockade. Tumor-reprogrammed macrophages with high purine metabolism exhibit decreased gene expression levels related to phagocytosis and antigen presentation while displaying elevated levels of immunosuppressive and angiogenic genes 24. While granulocyte-macrophage colony-stimulating factor (GM-CSF)-dependent macrophages exhibit typical M1 type high stimulatory activity, macrophage colony-stimulating factor (M-CSF)-dependent macrophages adopt an M2 phenotype. This M2 phenotype can result from the purinergic pathway, which directs the release of extracellular ATP and its conversion to adenosine through co-expressed exonucleotidases CD39 and CD73. Adenosine drives these cells toward the M2 state and increases the secretion of EMT-related cytokines, such as IL-1β, IL-6, IL-10, and VEGF 21.

### Aerobic metabolism

ROS, an aerobic metabolite of cells, is significantly elevated in macrophages and gastric adenocarcinoma cells compared to normal cells 25. ROS secreted by macrophages can activate the TNF-α/NF-κB, hypoxia inducible factor (HIF)-1α, TGF-β/Smad, PI3K/AKT/glycogen synthase kinase 3 beta (GSK3β)/β-catenin, nuclear factor erythroid 2-related factor 2 (Nrf2)/Notch1, or ERK signaling pathways, leading to the upregulation of activator protein 1 (AP-1), MMPs, urokinase-type plasminogen activator (uPA), and urokinase-type plasminogen activator receptor (uPAR). Consequently, this triggers the EMT of tumor cells 26.

### Autophagy

Autophagy, a key catabolic process, significantly impacts the generation, function, and polarization of macrophages 27. In monocytes and TAMs, autophagy facilitates monocyte-macrophage differentiation, promotes M2 polarization, enhances tumor-promoting activities, and impairs phagocytosis and antigen presentation, thereby potentiating EMT and metastasis of tumor cells 28.

Tumor cells induce autophagy in monocytes and TAMs by secreting cytokines such as M-CSF, GM-CSF, and IL-4. Specifically, M-CSF alone or GM-CSF combined with IL-4 induces autophagy in monocytes by activating C-Jun NH2-terminal kinase and blocking ATG5 cleavage, promoting M2 differentiation and preventing monocyte apoptosis 29, 30. Colon cancer-derived cathepsin S stimulates autophagy to induce M2 transformation of TAMs, promoting tumor development. Glioma-derived exosomes enriched in IL-6 and miR-155-3p initiate autophagy and promote M2 polarization in TAMs through the IL-6-p-STAT3-miR-155-3p-autophagy-p-STAT3 positive feedback loop, enhancing glioma progression 31. Autophagic TAMs secrete TGF-β1 *via* the fucosyltransferase 4 (FUT4)/p-ezrin pathway, inducing EMT in lung adenocarcinoma cells 32. In a mouse model of ovarian cancer with peritoneal metastasis, TIM4^+^ TAMs reduce mTORC1 activity through arginase-1-mediated arginine depletion, exhibiting higher mitochondrial OXPHOS and mitophagy activity, thus promoting ovarian cancer growth and peritoneal metastasis 33. In breast cancer, lysosome associated membrane protein type 2A (LAMP2a) on the lysosomal membrane of TAMs degrades peroxiredoxin 1 (PRDX1) and CREB-regulated transcription coactivator 1 (CRTC1) *via* chaperone-mediated autophagy (CMA), reducing ROS generation and activating M2 polarization and tumor-promoting activities 34.

Interestingly, inhibiting macrophage autophagy can also induce tumor cell EMT. Reduced autophagy levels in TAMs within the HCC microenvironment are linked to poor prognosis and increased microvascular metastasis. HCC suppresses macrophage autophagy initiation by upregulating mechanistic target of rapamycin (mTOR) and unc-51-like kinase 1 (ULK1) phosphorylation. The accumulation of the NLRP3 inflammasome due to autophagy inhibition in TAMs promotes the cleavage, maturation, and release of IL-1β, accelerating HCC metastasis by enhancing EMT. Additionally, autophagy inhibition triggers macrophage self-recruitment *via* the C-C motif chemokine ligand (CCL) 20/C-C chemokine receptor (CCR) 6 signaling axis, further contributing to HCC progression 35. This paradox highlights the complex role of autophagy in regulating TAMs.

## The regulation of tumor metabolic microenvironment on TAM-mediated EMT

**Figure 2:**
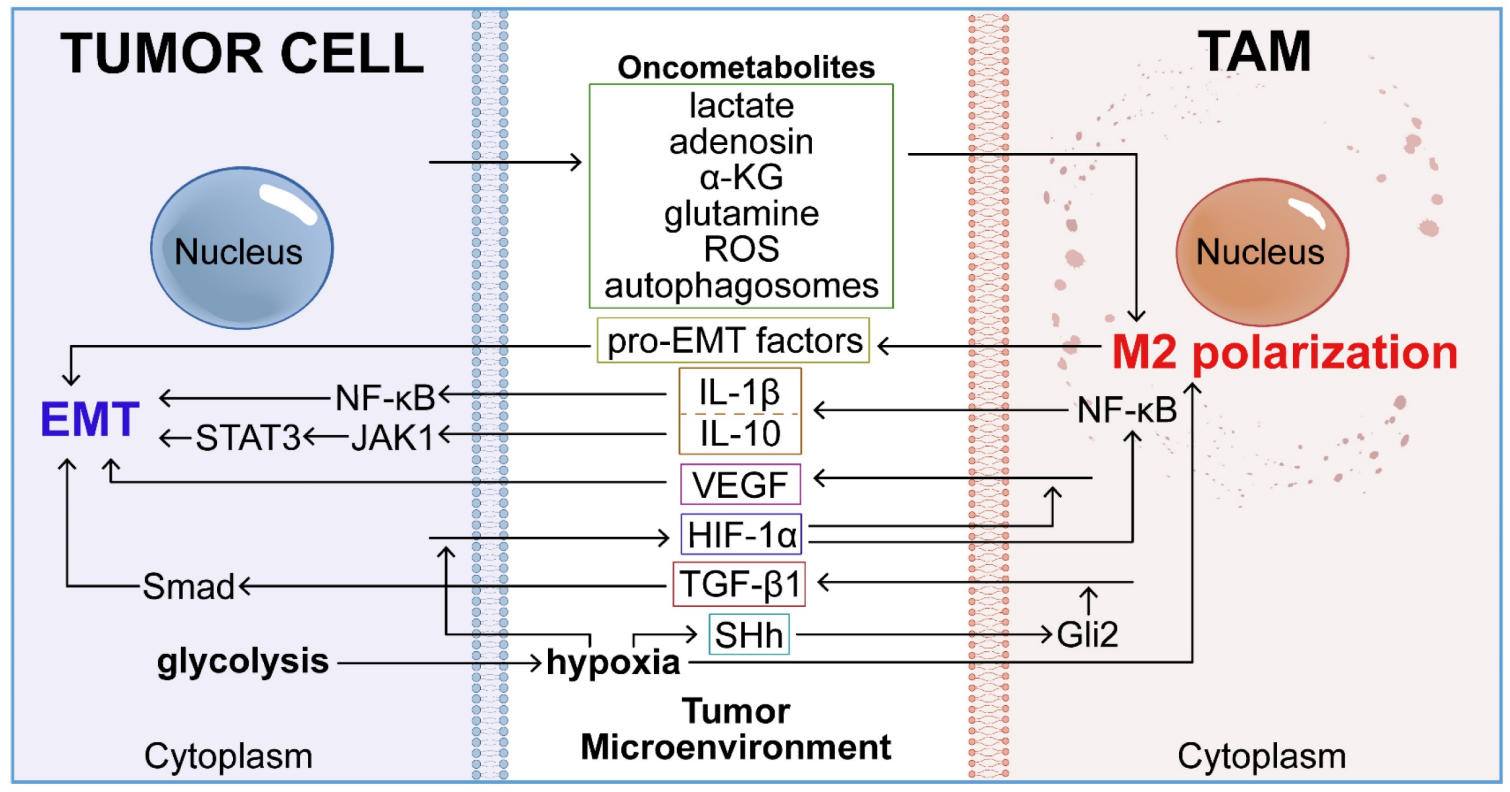
The distinct metabolic microenvironment of tumors profoundly influences TAM-mediated EMT. Elevated metabolic activities within this oncogenic setting—such as metabolite release (lactate, adenosine, α-ketoglutarate, glutamine, and reactive oxygen species), aerobic glycolysis, and autophagy—induce systemic biochemical shifts in the tumor. These changes in the TME promote M2 polarization of TAMs and trigger the secretion of EMT-related mediators (TGF-β1, IL-1β, IL-10, and VEGF). This cascade of events subsequently enhances the activation of EMT in tumor cells.

The elevated metabolic activities of tumor cells, including hypoxia 36, metabolite secretion 37, aerobic metabolism 26, and autophagy 38, define the unique metabolic landscape of the TME. TAMs, a resilient cell population, remain activated within this distinctive metabolic milieu shaped by tumor cells 39. This tumor-altered metabolic microenvironment profoundly affects macrophage functions, thereby facilitating EMT and promoting the metastatic progression of tumor cells (**Figure 2**).

### Tumor cell-induced hypoxia

Under aerobic conditions, normal cells produce ATP primarily through OXPHOS, while glycolysis predominantly generates ATP in the absence of oxygen. However, the rapid proliferation of tumor cells increases their energy demands, leading to the preferential use of glycolysis even in the presence of oxygen, a phenomenon known as the Warburg effect or aerobic glycolysis 13. The combination of insufficient blood supply due to tumor growth and aerobic glycolysis can result in hypoxia 36. M2 macrophages are more abundant in the hypoxic regions of solid tumors compared to M1 macrophages. The migration of macrophages to these hypoxic regions is driven by soluble factors secreted by the tumor into the circulation, including VEGF, endothelial monocyte-activating polypeptide II (EMAPII), endothelin-2, C-X-C chemokine receptor 4 (CXCR4), and semaphorin3A 40. Hypoxia in the TME can stimulate the secretion of HIF-1α by tumors and the release of EMT-promoting factors by macrophages.

#### Hypoxia-induced HIF-1α

TME hypoxia induces tumor cells to secrete the hypoxia-responsive transcription factor HIF-1α. HIF-1α influences both tumor cells and TAMs, stimulating the expression of EMT-related factors such as VEGF 41, histone deacetylase 3 (HDAC3) 42, AXL 43, membrane-type 4 matrix metalloproteinase (MT4-MMP) 44, MMP-9 36, lysyl oxidase (LOX) 5, COX-2 18, Twist, Snail1/2, and Zeb1 42, and activating the Notch 5 and Hedgehog (Hh) 42 pathways. These actions collectively promote EMT in tumor cells. Additionally, hypoxic exosomes derived from pancreatic cancer cells can induce macrophages to adopt an M2 phenotype in a manner dependent on HIF-1α or HIF-2α, further promoting EMT in tumor cells 19.

#### Hypoxia-induced EMT-related factor secretion of macrophages

Tumor cell-induced hypoxia enhances the secretion of EMT-promoting factors such as TGF-β1, IL-1β, IL-10, and VEGF by macrophages.

TGF-β1 is a vital regulator of EMT. Tumor-induced hypoxia elevates glioma-associated oncogene homolog (Gli2) expression in TAMs through the Sonic Hedgehog (SHh) signaling pathway, inducing M2 polarization of TAMs and increasing TGF-β1 secretion 45. Hypoxia-induced necrotic fragments of cancer cells, like HCC cells, and HIF-1α secreted by hypoxic tumor cells recruit TLR4 to the macrophage cell membrane, activating the toll-like receptor (TLR) 4/TIR-domain-containing adaptor-inducing interferon-β (TRIF)/NF-κB pathway. This activation upregulates IL-1β and IL-10 expression in macrophages, inducing EMT in breast cancer, pancreatic cancer, HCC, and others 46, 47. Hypoxic TAMs are a rich source of VEGF, significantly enhancing tumor angiogenesis, exacerbating hypoxia, and promoting EMT 40. Under hypoxic conditions, HIF-1α/2α triggers the upregulation of VEGF-A expression in TAMs, thereby promoting TAM-induced angiogenesis and metastasis 41.

### Tumor acidic metabolites

Aerobic glycolysis in tumor cells produces substantial hydrogen ions and acidic metabolites, creating an acidic microenvironment 37. In human HCC, macrophages express high levels of carbonic anhydrase XII (CA12). A transient glycolytic activation triggers sustained CA12 expression on tumor-infiltrating monocytes and macrophages *via* autocrine cytokines and HIF-1α signaling pathways. CA12 enables macrophage survival in the acidic TME and stimulates the production of large amounts of CCL8, facilitating tumor EMT and metastasis 48. High glycolytic activity in melanoma cells leads to pronounced acidification of the TME, which induces inducible cAMP early repressor (ICER) expression in TAMs, resulting in M2-type polarization and promoting tumor growth 49.

Lactate, an acidic metabolite from tumors, stimulates TAM polarization to the M2 phenotype, promoting tumor EMT and metastasis. In a lactate-rich TME, GM-CSF-activated macrophages undergo M2 polarization and produce large amounts of anti-inflammatory cytokines 37.

Lactate generated within tumor cells interacts with GPR132 50, promotes TGF-β and VEGF expression, and induces M2 polarization in TAMs *via* HIF-1α activation, thereby promoting tumor growth 51, 52. Additionally, lactate stimulates histone lysine lactylation in M1 macrophages, triggering the expression of M2-like homeostatic genes such as VEGF and Arg1, and promoting the transition to the M2 phenotype 53. Tumor-derived lactic acid induces macrophage M2 polarization by activating the ERK/STAT3 pathway in breast cancer and the MCT/HIF-1α pathway in gastric cancer 54, 55. Lactate also induces TAM-like phenotypes in macrophages and stimulates CCL5 secretion *via* Notch signaling. CCL5 promotes aerobic glycolysis in breast cancer cells by modulating AMP-activated protein kinase (AMPK) signaling, further inducing EMT and migration 56. Lactate secreted by HCC and PDAC cells increases ROS levels in macrophages, inducing M2 polarization and upregulating VEGF expression through Nrf2 activation. In turn, M2 macrophages activate the Nrf2 signaling pathway in cancer cells *via* paracrine VEGF secretion, inducing tumor EMT 57.

### Autophagy in tumor cells

Autophagy in tumor cells can reprogram monocytes and TAMs through various mechanisms, such as secreting autophagosomes, promoting monocyte-to-macrophage differentiation, and reducing the phagocytic and cytotoxic properties of TAMs 58, thereby driving tumor cell EMT. Tumor cell-released autophagosomes (TRAPs) can convert macrophages into an M2-like phenotype *via* the TLR4/MyD88/p38/STAT3 pathway, subsequently suppressing T cell activation 59. Interferon-gamma (IFN-γ)-induced autophagy in tumor cells and CSCs leads to the production of arginine resynthesis precursor asymmetric dimethylarginine (ADMA). ADMA impacts macrophages by delaying phagocytosis, reducing proliferation, and NO production, and inducing M2 polarization, thus promoting tumor progression 1. Inhibition of autophagy in triple-negative breast cancer (TNBC) has been shown to promote ROS-dependent macrophage migration inhibitory factor (MIF) secretion and stimulate M1 polarization 60. Furthermore, M2 TAMs can promote EMT by suppressing autophagy in tumor cells. For instance, renal cell carcinoma (RCC) can induce M2-type macrophages to secrete CCL2, inhibiting muscleblind-like protein 2 (MBNL2)/B-cell lymphoma 2 (Bcl-2)/beclin 1-mediated autophagy in RCC cells, leading to cell growth and EMT 61.

### Other oncometabolites

Beyond acidic metabolites secreted by tumor cells, macrophage reprogramming within the TME is influenced by various oncometabolites such as adenosine, glutamine, α-KG, and ROS. These oncometabolites drive monocyte recruitment, promote M2 macrophage polarization, and inhibit M1 activation, thereby facilitating EMT.

Adenosine promotes M2 macrophage polarization and monocyte migration towards tumor cells 10. Activation of the Adenosine A2A receptor reduces TNF-α, IL-6, and IL-12 production in macrophages while increasing IL-10, VEGF, and IL-1β levels 21, 62, 63. Similarly, the A2B receptor decreases TNF-α and IL-12 production and enhances IL-6 and IL-10 production in macrophages 21.

α-KG is a crucial regulator of M2 macrophage activation, enhancing FAO and inducing epigenetic reprogramming of gene expression in M2 macrophages 64. Additionally, α-KG inhibits M1 activation by upregulating the NF-κB pathway, destabilizing HIF-1α, and accelerating glutamine decomposition, promoting M2 polarization 10.

Hypermetabolic cancer cells utilize ROS regulation, mediated by cell-cell interactions, to select macrophages that enhance their survival and malignancy 25. Tumor-derived ROS facilitate macrophage recruitment and M2 polarization, with macrophages reciprocally increasing ROS secretion, ultimately promoting tumor metastasis 26. ROS also activates the transcription factor aryl hydrocarbon receptor (AHR), further recruiting monocytes and activating macrophages 65. Non-small cell lung carcinoma (NSCLC) activates the ROS/PI3K/AKT axis, releases cytokines such as VEGF-C, CCL7, and IL-8 to recruit macrophages, and upregulates M-CSF expression to drive M2 polarization, thereby facilitating NSCLC progression 66.

## TAM-mediated glycolysis-dependent EMT in tumor cells

Aerobic glycolysis in tumor cells induces EMT through various intracellular pathways. TAMs further enhance this process by secreting cytokines that reprogram the aerobic glycolysis of tumor cells, thereby promoting EMT.

### The mechanism of glycolysis-dependent EMT in tumor cells

Aerobic glycolysis is associated with various cellular processes, including EMT, angiogenesis, hypoxia, lactate production, macrophage polarization, and T-cell activation 53. Pathway enrichment analysis has shown significant enrichment of EMT-related signaling pathways in patients with elevated glycolytic activity 67. The metabolic shift from OXPHOS to glycolysis in tumor cells enhances EMT by upregulating EMT-inducing transcription factors (EMT-TFs), suppressing E-cadherin, promoting MMP secretion, and inducing cytoskeleton remodeling. Glycolytic enzymes such as glucose-6-phosphate isomerase/autocrine motility factor (PGI/AMF) and alpha-enolase (ENO1) play pivotal roles in activating EMT through the regulation of EMT-TFs 68.

### TAMs induce glycolysis-dependent EMT in tumor cells by secreting cytokines

**Figure 3:**
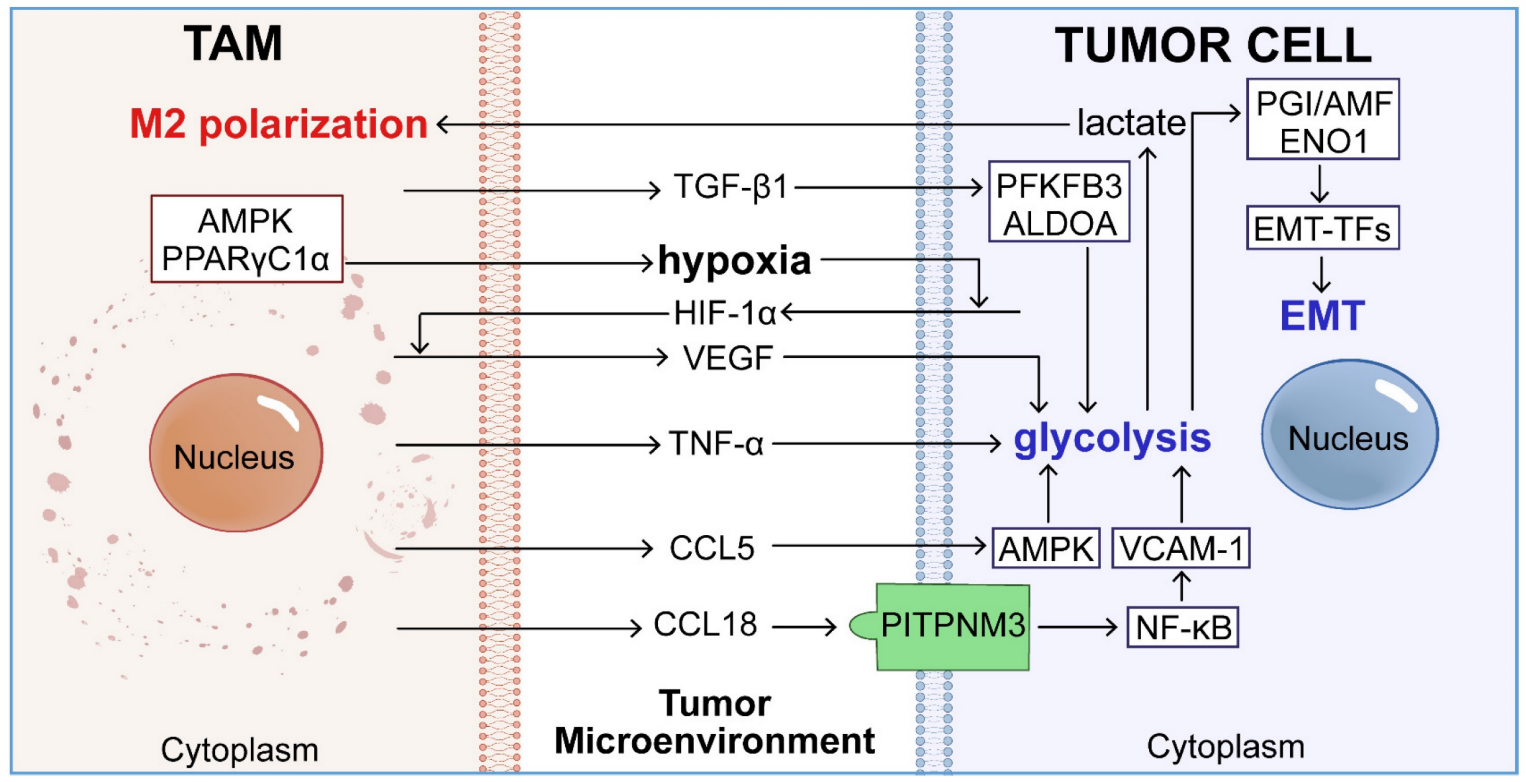
The roles of TAMs in glycolytic-driven EMT. Activated macrophages secrete key cytokines, including CCL5, CCL18, VEGF, TNF-α, and TGF-β, which interact with various signaling pathways to stimulate glycolysis within tumor cells. This promotion of glycolysis leads to elevated transcription of EMT-TFs through glycolytic enzymes such as PGI/AMF and ENO1, thereby intensifying the EMT process and contributing to tumor metastasis.

TAMs can reprogram the aerobic glycolysis of tumor cells by secreting cytokines like tumor necrosis factor TNF-α, chemokines CCL5 and CCL18, and growth factors VEGF and TGF-β1 69, thereby inducing EMT (**Figure 3**).

#### Tumor necrosis factor

Pancreatic cancer cells exhibit a high glycolytic activity, characterized by overexpression of glycolytic enzymes and increased lactate production, partially driven by TAMs 68. TAMs enhance their own AMPK and peroxisome proliferator-activated receptor-gamma coactivator 1α (PPARγC1α) to promote tumor hypoxia while releasing TNF-α to boost glycolysis in tumor cells 19.

#### Chemokine

CCL5 secreted by TAMs activates AMPK signaling in breast cancer cells, promoting aerobic glycolysis, thereby inducing EMT and migration 56. M2 TAMs highly express CCL18, which interacts with PITPNM3 in PDAC cells to activate the NF-kappaB (NF-κB) pathway, resulting in overexpression of vascular cell adhesion molecule-1 (VCAM-1). Elevated VCAM-1 further enhances aerobic glycolysis and lactate secretion in PDAC cells, facilitating macrophage M2 polarization and PDAC progression 70.

#### Growth factor

M2 TAM-derived exosomal metastasis-associated lung adenocarcinoma transcript 1 (MALAT1) induces aerobic glycolysis in gastric cancer cells by activating the β-catenin and HIF-1α signaling pathways, enhancing their proliferation, metastasis, and chemoresistance in a glycolysis-dependent manner 71. Glycolytic cancer cells can induce TAMs to express VEGF in a HIF-1α-dependent manner and promote M2 polarization *via* lactate secretion, further enhancing aerobic glycolysis in PDAC cells 68. TGF-β1 secreted by TAMs stimulates glycolysis and enhances PANC-1 cell invasion by upregulating the expression of 6-phosphofructo 2-kinase/fructose 2, 6-bisphosphatase 3 (PFKFB3) and the glycolysis gene aldolase A (ALDOA) 68. Additionally, TGF-β secreted by M2-type TAMs promotes glycolysis in bladder cancer cells *via* the Smad2/3 pathways, further driving EMT 52.

## Targeting metabolic interactions between tumors and TAMs for metastatic tumor therapies

**Figure 4:**
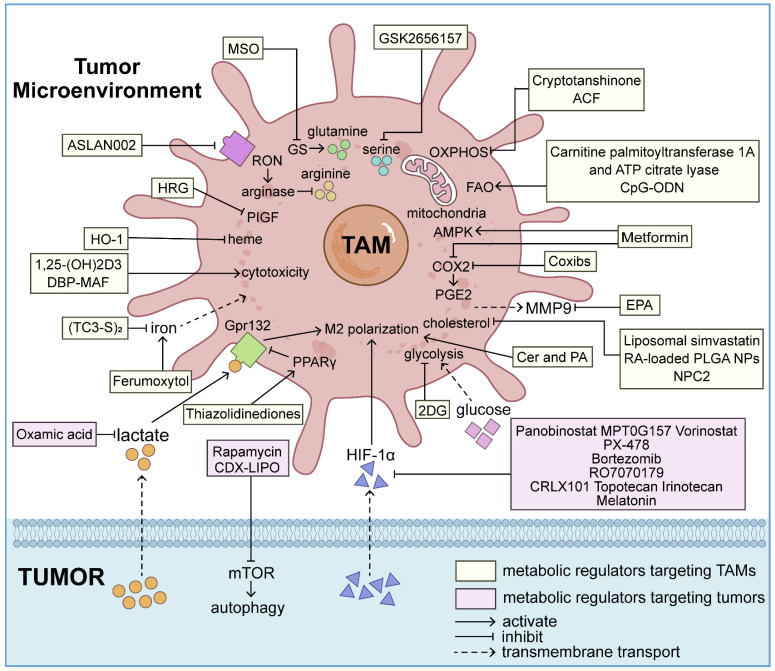
A variety of antitumor therapeutic modalities targeting metabolic processes in both TAMs and tumor cells have emerged to combat invasive and metastatic malignancies. These treatments reprogram TAMs from a tumorigenic state to an anti-tumor state. Simultaneously, metabolic regulators aimed at cancer cells and their bioproducts can significantly modify the TME, indirectly reducing the EMT-inducing potential of TAMs. These therapeutic strategies offer pathways to inhibit EMT, thereby decelerating tumor progression.

The inhibition of tumor metastasis by blocking EMT has consistently captured the research community's interest, with a growing focus on the intricate interplay between TAMs and tumor cells. Numerous research institutions are currently conducting clinical studies on the metabolic reprogramming of macrophages and tumor cells to treat aggressive tumors (**Figure 4**).

### Targeting metabolism of TAMs

Tumor-induced metabolic reprogramming of TAMs enhances their pro-tumorigenic capabilities, including promoting EMT and metastasis. Targeting glucose 13, lipid 72, amino acid 73, mitochondrial 38, iron 74, vitamin 74, heme metabolism 75, and the AMPK metabolic pathway 76 in macrophages presents promising therapeutic strategies for augmenting antitumor activities and inducing TAM M1 polarization (**Table 1**).

#### Glucose metabolism

Inhibition of macrophage glycolysis by the competitive inhibitor hexokinase II (HK2), 2-deoxy-D-glucose (2DG), reduces the production of pro-EMT factors, such as IL-10, M-CSF, and MMP-9, facilitating M1 polarization of TAMs and reversing TAM-mediated angiogenesis, PDAC cell extravasation, and EMT 13, 77. The 18F-fluoro-2DG (18F-FDG) radiopharmaceutical is widely employed in Positron Emission Tomography/Computed Tomography (PET/CT) examinations 78.

#### Lipid metabolism

Drugs targeting lipid metabolism are demonstrating efficacy in anti-tumor therapy. Targeting FAO, lipids, cholesterol, and COX2 can reverse macrophage M2 polarization by modulating lipid metabolism in macrophages, thereby inhibiting tumor progression.

##### Targeting FAO

Targeting peroxisome proliferator-activated receptor-gamma (PPARγ), PPARγ-coactivator-1β (PGC-1β), or STAT6 effectively reverses macrophage M2 polarization by inhibiting the FAO pathway. Combining FAO with fatty acid synthesis enhances macrophage antitumor activity 10. Carnitine palmitoyltransferase 1A and ATP citrate lyase increase macrophage antitumor activity by promoting the integration of FAO and lipid biosynthesis 79.

Intratumoral injection of TLR agonists increases monocyte recruitment and infiltration, inducing M1 polarization of M2 TAMs 74, 80. The TLR9 agonist cytidine-phosphate-guanosine oligodeoxynucleotide (CpG-ODN) effectively activates de novo lipid biosynthesis by promoting the shunting of macrophage FAO and TCA cycle intermediates, thereby inducing macrophage phagocytosis of CD47^+^ cancer cells 79. CpG-ODN is now recognized as the most potent immunostimulant among known vaccine adjuvants 81.

##### Targeting lipids

Exogenously applied lipids and lipid analogs have demonstrated anti-tumor activity in several cancers. The combination of natural lipids ceramide (Cer) and palmitic acid (PA) induces macrophage M1 polarization and inhibits M2 macrophage-driven EMT in breast cancer and colorectal cancer 82, 83. Additionally, an extra-pure formulation of eicosapentaenoic acid (EPA), an omega-3 polyunsaturated fatty acid, as free fatty acid (EPA-FFA), prevents colon cancer EMT and progression in colitis-associated cancer (CAC) by counteracting macrophage-derived MMP-9 upregulation of Notch1 signaling 84.

##### Targeting cholesterol

Targeting cholesterol metabolism in TAMs can promote M1 polarization and reverse tumor cell EMT. High cholesterol levels in the TME promote greater infiltration of M2 macrophages in breast cancer 85.

Simvastatin, delivered *via* liposomes, restores sensitivity to paclitaxel in EMT-associated drug-resistant NSCLC cells by inhibiting the cholesterol/lipid raft/integrin β3/FAK pathway. Simvastatin also promotes TAM M1 polarization and suppresses EMT through its negative regulation of the cholesterol-related liver X receptor (LXR)/ATP-binding cassette protein A1 (ABCA1) pathway 72.

Cancer cells' high metabolic activity can amplify IL-4 receptor activity by upregulating macrophage cholesterol efflux transporters, shifting TAMs towards an M2 phenotype. A poly(lactic-co-glycolic acid) (PLGA) nanoparticle (NP)-based drug delivery system loaded with retinoic acid (RA) and coated with cholesterol (CHO) can impede colorectal cancer cell EMT and M2 polarization. The CHO coating enhances membrane fusion capability and directs NPs towards M1 polarizing signals by suppressing cholesterol efflux and retinoid X receptors in TAMs 86.

Premalignant lung adenomas recruit immature macrophage-lineage cells (IMCs) to their stroma through CCR1-mediated pathways, promoting EMT. The recruitment of IMCs is impeded by the cholesterol-binding protein Niemann-Pick type C2 (NPC2), which regulates cholesterol trafficking from the late endosomes/lysosomes to the cytosol. Exogenous NPC2 decreases cholesterol levels in IMCs, suppressing CCR1 ligand CCL6 secretion 87.

##### Targeting PGE2

TAMs facilitate tumor metastasis by responding to lipid metabolites secreted by cancer cells or TAMs, particularly PGE2. Nonsteroidal anti-inflammatory drugs (NSAIDs) like coxibs (celecoxib, rofecoxib, valdecoxib) 88, etodolac 89, and NS-398 88, are notable cancer prevention agents that inhibit COX-2 by elevating ROS concentrations, thereby reducing cancer recurrence. Celecoxib effectively reduces PGE2 expression in bladder cancer and promotes M2 polarization of TAMs in colon cancer 90, 91. Additionally, metformin inhibits TAM infiltration by suppressing the COX2/PGE2 axis, thus slowing prostate cancer progression 92.

#### Amino acid metabolism

TAMs possess a high catabolic capacity for arginine and tryptophan, which are essential for T cell anti-tumor activity. Replenishing these nutrients by suppressing arginase, indoleamine 2, 3-dioxygenase (IDO), and nitric oxide synthase can enhance T cell survival and function 74. Recepteur d'Origine Nantais (RON) signaling in macrophages promotes macrophage spreading, phagocytosis, and M2 polarization by stimulating arginase expression and reducing responses to pro-inflammatory stimuli like IFN-γ and LPS 74. In breast cancer, the RON/MET receptor tyrosine kinase inhibitor BMS-777607/ASLAN002 increases the abundance of pro-inflammatory macrophages and reduces lung metastasis 93.

Glutamine catabolism in macrophages stimulates M2 polarization. Methionine sulfoximine (MSO) inhibits glutamine synthase (GS), diverting glucose rather than glutamine into the TCA cycle, inducing M1 polarization and preventing lung carcinoma metastasis 94. The PERK inhibitor GSK2656157 suppresses macrophage serine biosynthesis and immunosuppressive activity, delaying melanoma growth 22. Histidine-rich glycoprotein (HRG) induces M1 polarization of TAMs and promotes vascular normalization by downregulating placental growth factor (PLGF) 73.

#### Mitochondrial metabolism

Treatment of M2 TAMs with the immunomodulator cryptotanshinone activates apoptosis signal-regulating kinase 1 (ASK1), subsequently stimulating autophagy and promoting M1 polarization. This process impairs mitochondrial OXPHOS and fusion in M2 macrophages, reduces their oxygen consumption rate, and stimulates NO and ROS production, thereby inhibiting the proliferation and motility of TNBC cells 38. *In vitro* studies have shown that the antibacterial agent and OXPHOS metabolic inhibitor acriflavine (ACF) reduces pancreatic cancer EMT and invasion, and shifts macrophages to a M1-like phenotype 95.

#### Iron metabolism

The divergent iron metabolism in M1 and M2 macrophages is notable. M1 macrophages exhibit heightened ferritin expression, promoting intracellular iron retention. In contrast, M2 macrophages show increased ferroportin expression, promoting iron efflux and providing tumor cells with the iron necessary for proliferation 74. The intracellular iron chelator (TC3-S)_2_ has been shown to reverse the iron-processing function of M2 macrophages from iron release to sequestration, blocking their tumor-promoting effects 96. Additionally, external iron supplementation with ferumoxytol has demonstrated therapeutic efficacy in slowing the metastasis of early mammary cancers by stimulating M1 macrophage polarization and ROS production 97.

#### Vitamin metabolism

Vitamin D influences the tumor-promoting and anti-tumor activities of macrophages through various mechanisms. Vitamin D-1-hydroxylase CYP27B1, expressed at lower levels in TAMs, catalyzes the conversion of 25-hydroxyvitamin D (25D) to 1, 25-dihydroxyvitamin D3 [1, 25-(OH)2D3]. Macrophages secrete the host-defense peptide cathelicidin, which effectively lyses proliferating B cell lymphoma cells. Stimulation of M2 macrophages with 1, 25-(OH)2D3 restores cathelicidin production and cytotoxicity against B-cell lymphoma 98. Overexpression of the Vitamin D receptor (VDR) can prevent EMT in breast cancer cells co-cultured with macrophages. In breast cancer cells, macrophage-secreted TNF-α inhibits VDR expression. Administration of calcitriol [1, 25-(OH)2D3] mitigates macrophage-induced EMT and metastasis of breast cancer cells by preserving VDR 99. Vitamin D-binding protein-derived macrophage-activating factor (DBP-MAF) stimulates macrophages, downregulates vimentin expression, and reverses EMT in breast cancer cells 100.

#### Heme metabolism

Heme oxygenase-1 (HO-1), a key metabolic enzyme for heme degradation, restricts the differentiation and polarization of TAMs. The HO-1-derived catalytic product, carbon monoxide (CO), from TAMs, enhances mitochondrial activity in cancer cells, leading to increased E-cadherin levels and inhibiting EMT and prostate cancer progression 75. Therefore, therapeutic strategies targeting TAM modulation *via* HO-1 could yield significant anti-tumor effects.

#### AMPK metabolic pathway

AMPK, a serine/threonine protein kinase, functions as a central metabolic sensor in cellular energy homeostasis 101. Metformin enhances AMPK activity in macrophages, reduces STAT3 phosphorylation, and promotes mTOR suppression, thereby slowing monocyte transformation into macrophages, reducing TAM infiltration, and inhibiting M2 polarization 76, 102, 103. Metformin's influence on the AMPK pathway in macrophages decreases the expression of several genes in PDAC involved in ECM remodeling (including MMPs) and EMT 76. However, the specific regulatory mechanisms of metformin on macrophage metabolism *via* the AMPK pathway remain unclear.

### Targeting metabolism of tumor cells

Metabolic alterations within tumor cells can induce macrophage recruitment, M2 polarization, and secretion of EMT-related factors. Targeting metabolic pathways such as hypoxia, metabolite secretion, and autophagy in tumor cells can reverse macrophage manipulation by the metabolic microenvironment, effectively preventing cancer EMT and progression (**Table 2**).

#### Targeting tumor hypoxia

TME hypoxia induces HIF-1α secretion in tumor cells, regulating EMT by enhancing TAM functions. HDAC inhibitors (including panobinostat, MPT0G157, vorinostat), the HIF-1α inhibitor PX-478, proteasome inhibitor bortezomib 104, antisense oligonucleotide (ASO) RO7070179 (EZN-2968), camptothecin and its analogs (including CRLX101, topotecan, irinotecan), and melatonin 105 inhibit HIF-1α expression through various strategies. Additionally, exosomes derived from M1 macrophages can be engineered to express catalase in the membrane or carry DNA damage repair inhibitors, effectively alleviating tumor hypoxia and enhancing tumor DNA damage 106.

#### Targeting tumor metabolites

Specific targeting of the tumor metabolite lactate is currently being explored in preclinical cancer models. Lactate recognition by the G-protein coupled receptor Gpr132 on TAMs promotes M2 polarization, while PPARγ transcription factor suppresses Gpr132 expression 74. Inhibition of lactate production by pretreating MDA-MB-231 cells with the lactate dehydrogenase inhibitor oxamic acid can shift the polarization of tumor-derived GM-CSF-stimulated macrophages towards a pro-inflammatory phenotype 37.

PPARγ agonists, such as thiazolidinediones, or siRNA silencing of Gpr132 have shown efficacy in rendering TAMs insensitive to lactate stimulation, significantly impeding breast cancer progression 107.

#### Targeting tumor cell autophagy

Combining the autophagy activator rapamycin with hydroxychloroquine (HCQ) reduces macrophage M2 polarization and lowers CD47 and SIRPα expression levels in tumor cells and macrophages. This enhances macrophage phagocytic ability and increases glioblastoma (GBM) sensitivity to immune checkpoint inhibitors (ICIs). Rapamycin also reverses M2 macrophage-induced autophagy inhibition and EMT in RCC cells 61, 108. Rapamycin has been approved by the FDA for managing advanced RCC 109. Liposomal honokiol and the disulfiram/copper codelivery system (CDX-LIPO) significantly stimulate autophagy in GBM cells by interfering with the mTOR pathway, inhibiting aerobic glycolysis and lactic acid production, thereby converting M2 TAMs to the M1 type 110.

## Conclusions and Outlooks

Understanding and studying the effects of metabolic pathways that connect and diversify TAMs and tumor cells on EMT is essential for identifying potential TAM-associated immunotherapy targets and enhancing therapeutic efficacy of metastatic tumors. This review elucidates the metabolic crosstalk between TAMs and tumor cells within the TME, presenting a novel approach for immunotherapy of metastatic tumors.

Despite these advancements, several obstacles and challenges remain in developing and clinically implementing anti-tumor drugs targeting TAMs or tumor cell metabolism. Innovative research methodologies and strategies may provide solutions to these issues. This prospective analysis summarizes the existing challenges and potential strategies to overcome them.

### Identification and selection of metabolic therapeutic targets for TAMs or tumor cells

Tumors and TAMs exhibit a vast and diverse array of metabolic pathways and gene expression profiles, with significant variations in cellular metabolic states observed among different patients. One of the primary challenges in the clinical application of metabolic-related treatments is identifying metabolic targets that can be broadly applied across patients with various cancers, while also developing personalized treatment strategies based on individual metabolic profiles.

Investigating the metabolic reprogramming of TAMs across different cancer types and disease stages is a prevalent research strategy, utilizing techniques such as single-cell sequencing, transcriptome sequencing, gene enrichment analysis, qPCR, and metabolomics 111-113. The integration of these emerging research technologies allows for a comprehensive understanding of the alterations in TAMs' metabolic genes and their role in tumor metastasis on a patient-by-patient basis. This approach can identify key metabolic regulatory genes and metabolites that are applicable to populations with distinct cancer types. Additionally, it supports the development of personalized treatment strategies targeting the metabolism of patients with diverse cancers.

### The safety considerations for TAMs or targeted metabolic therapy in tumor cells

Despite normal cells from different tissues exhibiting varied metabolic profiles, cancer cells also show significant metabolic diversity and plasticity. However, few metabolic characteristics consistently distinguish cancer cells from normal cells 114. Both normal and tumor tissues produce numerous metabolites, and the same metabolite can play diverse roles in different tissues or organs 115. Therefore, precisely targeting TAMs or tumor cells with metabolic regulators and minimizing adverse effects on normal tissue metabolism presents a substantial challenge for the clinical application of this anti-tumor strategy.

Developing meticulously targeted metabolic regulators that specifically engage TAMs or tumor cells and circumvent the crucial metabolic products in normal cells is essential. This issue might be addressed by utilizing exosomes as targeted drug carriers to TAMs or tumor cells. Exosomes, natural drug delivery vehicles, can be genetically engineered and chemically modified to enhance their targeting capacity, thereby mitigating medication side effects 116. Other strategies based on small molecules, ligands, peptides, antibodies, and cells for targeted drug delivery also exist and can help mitigate the off-target effects of drugs 117.

### The dynamic fluctuations of TAMs or tumor cell metabolites

The abundance of cellular metabolites fluctuates dynamically, heavily influenced by the nutritional environment and cellular context of cancer cells 115. This variation spans both temporal and spatial dimensions. Therefore, a comprehensive understanding of the changing patterns of tumor metabolites across different cancer types and stages is essential. Additionally, metabolic modulators should be assessed through experimental pharmacokinetic trials to determine the optimal dosage, timing, and interval for administration that would best disrupt the pro-tumor metabolic interplay between TAMs and tumor cells.

Currently, various detection techniques enable the dynamic monitoring of metabolite levels in TAMs and tumor cells. Metabolomics, for example, involves the comprehensive quantification of diverse metabolites, including nutrients, drugs, signaling intermediates, and metabolic byproducts in blood, urine, tissue extracts, or other body fluids. Commonly employed metabolomics techniques include LC-MS, gas chromatography-mass spectrometry (GC-MS), and nuclear magnetic resonance (NMR) 118, 119. These techniques facilitate the real-time monitoring of changes in metabolites, aiding in the determination of optimal administration timing.

Given the spatial heterogeneity of tumor metabolites, it is necessary to study the distribution of therapeutic drugs and their corresponding metabolites in both tumor and normal tissues to ensure accurate targeting. Molecular spectral imaging (MSI) is an innovative technique for constructing molecular images, precisely depicting the distribution of drugs and metabolites within tissues and their substructures. The most extensively utilized ionization technique in MSI research is matrix-assisted laser desorption/ionization mass spectrometry (MALDI), which provides information about the penetration of drugs and metabolites into target organs 120.

In conclusion, this review systematically describes the metabolic symbiosis between TAMs and tumor cells in regulating tumor EMT, offering a novel perspective for the development of potential TAM- or tumor-targeted metabolic drugs. It lays the theoretical groundwork for formulating clinical treatment strategies for metastatic tumors and aids subsequent researchers in deciphering novel molecular mechanisms involved.

## Figures and Tables

**Table 1:** Targeting metabolism of TAMs

Drug	Research phase of the drug	Types of cancer	Drug-specific properties & Mechanism of inhibiting tumor cell EMT	References
2-deoxy-D-glucose (2DG)	Pre-clinical	Ehrlich ascites cancer, PDAC	HK2 competitive inhibitor; Target: glucose metabolism	PMID: 26597503 PMID: 27622062
Carnitine palmitoyltransferase 1A and ATP citrate lyase	Pre-clinical	PDAC	Enzymes; Target: lipid metabolism	PMID: 31813459 PMID: 30664738
Cytidine-phosphate-guanosine oligodeoxynucleotide (CpG-ODN)	Pre-clinical	PDAC	TLR9 agonist; Target: lipid metabolism	PMID: 30664738 PMID: 33831324
Ceramide (Cer) and palmitic acid (PA)	Pre-clinical	Colorectal cancer, breast cancer	Natural lipids; Target: lipid metabolism	PMID: 32222879 PMID: 35457057
Eicosapentaenoic acid (EPA)	Pre-clinical	Colon cancer	Omega-3 polyunsaturated fatty acid; Target: lipid metabolism	PMID: 26864323
Liposomal simvastatin	Pre-clinical	NSCLC	Liposome and statin based lipid-lowering drug; Target: cholesterol metabolism	PMID: 30662566
Retinoic acid-loaded PLGA nanocarriers	Pre-clinical	Colorectal cancer	Drug delivery system; Target: cholesterol metabolism	PMID: 36857852
Niemann-pick type C2 (NPC2)	Pre-clinical	Lung cancer	Cholesterol-binding protein; Target: cholesterol metabolism	PMID: 26183450
Celecoxib	FDA approval	Bladder cancer, colon cancer, Glioblastoma multiforme	NSAIDs and selective COX-2 inhibitors; Target: fatty acid metabolism (COX-2/PGE2 signal pathway)	PMID: 28096371 PMID: 21730361 PMID: 27693715
Rofecoxib	FDA approval	Glioblastoma multiforme		PMID: 27693715
Valdecoxib	FDA approval	Glioblastoma multiforme		PMID: 27693715
Etodolac	FDA approval	Gastrointestinal cancer		PMID: 12483244
NS-398	Pre-clinical	Glioblastoma multiforme		PMID: 27693715
Metformin	FDA approval	Pancreatic cancer, colorectal cancer, breast cancer, prostate cancer	Hypoglycemic drugs; Target: COX-2/PGE2 (fatty acid metabolism) and AMPK signal pathways	PMID: 26641266 PMID: 25552600 PMID: 35740547 PMID: 28157701 PMID: 30012567
ASLAN002/BMS777607	Phase I clinical trial (NCT01721148)	Breast cancer	RON/MET receptor tyrosine kinase inhibitor; Target: arginine (amino acid metabolism)	PMID: 23612011
Methionine sulfoximine (MSO)	Pre-clinical	Lung carcinoma	GS inhibitor; Target: GS (amino acid metabolism)	PMID: 28813676
GSK2656157	Pre-clinical	Melanoma	PERK inhibitor; Target: serine (amino acid metabolism)	PMID:35228694
Histidine-rich glycoprotein (HRG)	Pre-clinical	Fibrosarcoma, pancreatic adenocarcinoma	Plasma glycoprotein; Target: histidine (amino acid metabolism)	PMID: 21215706
Cryptotanshinone	Pre-clinical	TNBC	Immunomodulator; Target: mitochondrial metabolism	PMID: 35860009
Acriflavine (ACF)	Pre-clinical	PDAC	Antibacterial drug and metabolic inhibitor of OXPHOS; Target: mitochondrial metabolism	PMID: 32764982
(TC3-S)2	Pre-clinical	Breast cancer	Intracellular iron chelator; Target: iron metabolism	PMID: 27806101
Ferumoxytol	FDA approval	Early mammary cancer	Iron supplement; Target: iron metabolism	PMID: 28449873 PMID: 27668795
1,25-dihydroxyvitamin D3 [1,25-(OH)2D3]	FDA approval	Burkitt's lymphoma, breast cancer	Bioactive form of vitamin D; Target: vitamin metabolism	PMID: 25855493 PMID: 24821711
Vitamin D-binding protein-derived macrophage-activating factor (DBP-MAF)	Pre-clinical	Breast cancer	Macrophage-activating factor; Target: vitamin metabolism	PMID: 22213287
Heme oxygenase-1 (HO-1)	Pre-clinical	Prostate cancer	Metabolic enzyme; Target: heme metabolism	PMID: 26418896

**Table 2:** Targeting metabolism of tumor cells

Drug	Research phase of the drug	Types of cancer	Drug-specific properties & Mechanism of inhibiting tumor cell EMT	References
Panobinostat	FDA approval	Hodgkin lymphoma	HDAC inhibitors; Target: HIF-1α (tumor hypoxia)	PMID: 22408261
MPT0G157	Pre-clinical	Colorectal cancer		PMID: 26087180
Vorinostat	FDA approval	T-cell lymphoma		PMID: 17438089
PX-478	Phase I clinical trial (NCT00522652)	Prostate cancer, breast cancer, colorectal adenocarcinoma, pancreatic cancer	HIF-1α inhibitor; Target: HIF-1α (tumor hypoxia)	PMID: 18202012
Bortezomib	FDA approval	Multiple myeloma	Proteasome inhibitor; Target: HIF-1α (tumor hypoxia)	PMID: 14657528
RO7070179 (EZN-2968)	Phase I clinical trial (NCT02564614)	Advanced HCC	ASO; Target: HIF-1α (tumor hypoxia)	PMID: 30949444
CRLX101	Phase II clinical trial (NCT03531827)	Rectal cancer	Topoisomerase I inhibitor camptothecin and its analogs; Target: HIF-1α (tumor hypoxia)	PMID: 27784746
Topotecan	FDA approval	TNBC		PMID: 26623560
Irinotecan	FDA approval	Malignant glioma		PMID: 20066473
Melatonin	FDA approval	Prostate cancer	Hormonal drug; Target: HIF-1α (tumor hypoxia)	PMID: 21392092
Oxamic acid	Pre-clinical	Breast cancer	Lactate dehydrogenase inhibitor; Target: lactate (tumor metabolite)	PMID: 24823638
Thiazolidinediones	FDA approval	Breast cancer	PPARγ agonists Target: lactate (tumor metabolite)	PMID: 27692066
Rapamycin	FDA approval	GBM, RCC	Autophagy activator and mtor inhibitor; Target: autophagy (catabolism)	PMID: 32020472 PMID: 36244911
Liposomal honokiol and disulfiram/copper codelivery system (CDX-LIPO)	Pre-clinical	GBM	Liposome; Target: autophagy (catabolism)	PMID: 32817393

## Abbreviations

TAMs: Tumor-associated macrophages
TCA: Tricarboxylic acid
TME: Tumor microenvironment
EMT: Epithelial mesenchymal transition
MDR: Multidrug resistance
CSC: Cancer stem cell
OGT: O-GlcNAcylation transferase
PDEC: Pancreatic ductal epithelial cells
IL: Interleukin
TNF-α: Tumor necrosis factor alpha
PDAC: Pancreatic ductal adenocarcinoma
DNA: Deoxyribonucleic acid
OXPHOS: Oxidative phosphorylation
ROS: Reactive oxygen species
NO: Nitric oxide
PPP: Pentose phosphate pathway
NADPH: Nicotinamide adenine dinucleotide phosphate
iNOS: Inducible nitric oxide synthase
PFKFB3: 6-phosphofructo-2-kinase/fructose-2,6-biphosphatase 3
TGF-β: Transforming growth factor beta
HCC: Hepatocellular carcinoma
MAMs: Metastasis-associated macrophages
STAT: Signal transducer and activator of transcription
FAO: Fatty acid oxidation
NLRP3: NOD-like receptor thermal protein domain associated protein 3
VEGF: Vascular endothelial growth factor
MCP-1: Monocyte chemoattractant protein-1
MMP: Matrix metalloproteinase
COX-2: Cyclooxygenase 2
PG: Prostaglandins
PGE2: Prostaglandin E2
CTGF: Connective tissue growth factor
HGF: Hepatocyte growth factor
FGF: Fibroblast growth factor
NF-κB: NF-kappaB
ERK: Extracellular signal-regulated kinase
PI3K: Phosphatidylinositol-3-kinase
AKT/PKB: Protein kinase B
LPS: Lipopolysaccharide
Arg1: Arginase 1
PERK: Protein kinase RNA-like ER kinase
PSAT1: Phosphoserine aminotransferase 1
α-KG: Alpha-ketoglutarate
ATP: Adenosine triphosphate
c-Maf: Cellular musculoaponeurotic fibrosarcoma
UDP-GlcNAc: Uridine diphosphate N-acetylglucosamine
ACLY: Adenosine triphosphate-citrate lyase
GM-CSF: Granulocyte-macrophage colony-stimulating factor
M-CSF: Macrophage colony-stimulating factor
HIF: Hypoxia inducible factor
GSK3β: Glycogen synthase kinase 3 beta
Nrf2: Nuclear factor erythroid 2-related factor 2
AP-1: Activator protein 1
uPA: Urokinase-type plasminogen activator
uPAR: Urokinase-type plasminogen activator receptor
ATG5: Autophagy related 5
FUT4: Fucosyltransferase 4
mTORC1: Mechanistic target of rapamycin complex 1
LAMP2a: Lysosome associated membrane protein type 2A
PRDX1: Peroxiredoxin 1
CRTC1: CREB-regulated transcription coactivator 1
CMA: Chaperone-mediated autophagy
mTOR: Mechanistic target of rapamycin
ULK1: Unc-51-like kinase 1
CCL: C-C motif chemokine ligand
CCR: C-C chemokine receptor
EMAPII: Endothelial monocyte-activating polypeptide II
CXCR4: C-X-C chemokine receptor 4
HDAC: Histone deacetylase
MT4-MMP: Membrane-type 4 matrix metalloproteinase
LOX: Lysyl oxidase
Hh: Hedgehog
Gli: Glioma-associated oncogene homolog
SHh: Sonic Hedgehog
TLR: Toll-like receptor
TRIF: TIR domain containing adaptor molecule 1
CA12: Carbonic anhydrase XII
ICER: Inducible camp early repressor
MCT: Monocarboxylate channel transporter
AMPK: AMP-activated protein kinase
TRAPs: Tumor cell-released autophagosomes
IFN-γ: Interferon gamma
ADMA: Arginine resynthesis precursor asymmetric dimethylarginine
TNBC: Triple-negative breast cancer
MIF: Macrophage migration inhibitory factor
RCC: Renal cell carcinoma
MBNL2: Muscleblind-like protein 2
Bcl-2: B-cell lymphoma 2
AHR: Aryl hydrocarbon receptor
NSCLC: Non-small cell lung carcinoma
EMT-TFs: EMT-inducing transcription factors
PGI/AMF: Phosphoglucose isomerase/autocrine motility factor
ENO1: Enolase 1
PPARγC1α: Peroxisome proliferator-activated receptor-gamma coactivator 1α
VCAM-1: Vascular cellular adhesion molecule-1
MALAT1: Metastasis-associated lung adenocarcinoma transcript 1
ALDOA: Aldolase, fructose-bisphosphate A
HK2: Hexokinase II
2DG: 2-deoxy-D-glucose
18F-FDG: 18F-fluoro-2DG
PET/CT: Positron emission tomography/computed tomography
PPARγ: Peroxisome proliferator-activated receptor-gamma
PGC-1β: PPARγ-coactivator-1β
CpG-ODN: Cytidine-phosphate-guanosine oligodeoxynucleotide
Cer: Ceramide
PA: Palmitic acid
EPA: Eicosapentaenoic acid
FFA: Free fatty acid
CAC: Colitis-associated cancer
LXR: Liver X receptor
ABCA1: ATB-binding cassette protein A1
PLGA: Poly(lactic-co-glycolic acid)
NP: Nanoparticle
RA: Retinoic acid
CHO: Cholesterol
IMCs: Immature macrophage-lineage cells
NPC2: Niemann-pick type C2
NSAIDs: Nonsteroidal anti-inflammatory drugs
IDO: Indoleamine 2, 3-dioxygenase
RON: Recepteur d'origine nantais
MSO: Methionine sulfoximine
GS: Glutamine synthase
HRG: Histidine-rich glycoprotein
PLGF: Placental growth factor
ASK1: Apoptosis signal-regulating kinase 1
ACF: Acriflavine
1,25-(OH)2D3: 1,25-dihydroxyvitamin D3
VDR: Vitamin D receptor
DBP-MAF: Vitamin D-binding protein-derived macrophage-activating factor
HO-1: Heme oxygenase-1
CO: Carbon monoxide
ASO: Antisense oligonucleotide
HCQ: Hydroxychloroquine
GBM: Glioblastoma
ICIs: Immune checkpoint inhibitors
CDX-LIPO: Liposomal honokiol and disulfiram/copper codelivery system
qPCR: Quantitative real-time PCR
GC-MS: Gas chromatography-mass spectrometry
NMR: Nuclear magnetic resonance
MSI: Molecular spectral imaging
MALDI: Matrix-assisted laser desorption/ionization mass spectrometry
